# The association of vitamin D insufficiency with the prevalence of obesity in children: implications for serum calcium levels, alkaline phosphatase activity, and bone maturation

**DOI:** 10.3389/fnut.2024.1466270

**Published:** 2024-10-30

**Authors:** Yue Xu, Lingyun Song, Li Zhou

**Affiliations:** ^1^Department of Pediatrics, Hainan Hospital of PLA General Hospital, Sanya, China; ^2^Department of Endocrinology, Hainan Hospital of PLA General Hospital, Sanya, China; ^3^Department of Pediatrics, Lingshui Li Autonomous County People’s Hospital (Hainan Branch of the Affiliated Hospital of Qingdao University), Lingshui, China

**Keywords:** vitamin D, school-aged children, obesity, bone age, BMI

## Abstract

**Background:**

Vitamin D deficiency has been identified as a potential risk factor for various adverse health outcomes. However, its specific role in metabolic regulation and skeletal development in school-aged children is not fully understood. This study aimed to explore the correlation between vitamin D deficiency and childhood obesity rates, and its impact on serum calcium, alkaline phosphatase, and bone age in children.

**Methods:**

The study analyzed clinical data from 159 school-aged children who underwent medical examinations. Participants were divided into the 25-hydroxyvitamin D_3_ (25(OH)D_3_) deficiency group and the 25(OH)D_3_ normal group based on their serum levels. We compared body mass index (BMI), total cholesterol (TC), triglycerides (TG), Ca, ALP, bone age, fasting blood glucose (FBG), and hemoglobin A1c (HbA1c) between the two groups. Logistic regression and Spearman correlation analyses were performed to further investigate relationships between 25(OH)D_3_ levels and metabolic and bone-related markers.

**Results:**

This study showed that the 25(OH)D_3_ deficiency cohort exhibited significantly higher BMI, TC, TG, and ALP levels, with lower Ca levels and delayed bone age compared to the normal group. Logistic regression analysis identified Ca, ALP, and bone age as significant predictors of 25(OH)D_3_ deficiency. Subgroup analysis showed that in the 25(OH)D_3_ deficient group, children with higher BMI had elevated TC, ALP levels, and delayed bone age, while Ca levels were lower. Correlation analysis confirmed the predictive value of these markers for 25(OH)D_3_ deficiency.

**Conclusion:**

Our findings demonstrate that 25(OH)D_3_ deficiency is strongly associated with obesity in school-aged children and may negatively affect normal skeletal development. Regular monitoring of 25(OH)D_3_ levels in school-aged children is essential for ensuring proper growth and development, especially in those at risk for obesity.

## 1 Introduction

Vitamin D, a lipid-soluble vitamin, is integral to a myriad of physiological processes, notably enhancing cellular proliferation, facilitating differentiation, and modulating immune responses (1). As an essential nutrient not endogenously produced by the human body, vitamin D must be acquired through dietary intake or sunlight exposure, such as fish liver, egg yolks, and certain dairy products. Once absorbed in the small intestine, dietary sources of vitamin D are transported via chylomicrons into the bloodstream and converted in the liver into 25(OH)D_3_. This intricate physiological process is the principal form of vitamin D circulating in the bloodstream, serving a vital function in the facilitation of calcium absorption and bone mineralization.

Previous studies showed that infants, children, adolescents, and pregnant women require 400 IU/d of vitamin D, and inadequate dietary intake can lead to insufficiency in vitamin D, increasing the risk of conditions such as adult osteomalacia and childhood rickets (2–4).

Vitamin D insufficiency is a globally recognized public health challenge, affecting billions of individuals worldwide (5–7). In China, vitamin D deficiency poses an equally pressing concern, notably affecting school-aged children. Ongoing developmental processes in preschool-aged children may render them susceptible to a range of gastrointestinal disorders, potentially hindering efficient vitamin D absorption. Furthermore, selective eating behaviors in this age group increase their risk of vitamin D deficiency (8, 9). Research indicates that 34% of obese children are affected by this deficiency (10). Vitamin D deficiency has been alarmingly associated with a spectrum of severe health conditions, encompassing cardiovascular diseases, metabolic disorders, endocrine imbalances, and even childhood cancers (11). Overall, the issue of vitamin D insufficiency in school-aged children is increasingly gaining attention from various sectors of society.

TC, TG, FBG, and HbA1c are widely acknowledged in glucose and lipid metabolism for their diagnostic value. In clinical practice, the assessment of vitamin D status is conventionally conducted by detecting 25(OH)D_3_ levels. Therefore, we aim to assess how variations in 25(OH)D_3_ levels affect Ca levels, ALP activity, and bone age in this pediatric population. This study aims to investigate the risk of childhood obesity and foster optimal skeletal growth and development by addressing vitamin D deficiency in school-aged children. This study aims to examine childhood obesity and promote skeletal growth and development in school-aged children by addressing the deficiency of 25(OH)D_3_.

## 2 Materials and methods

### 2.1 Subjects and data collection

We retrospectively analyzed clinical data collected from 159 school-aged children (aged 6–12 years) during health examinations at the Hainan Hospital of PLA General Hospital and Lingshui Li Autonomous County People’s Hospital from April 2019 to April 2022. Baseline data were systematically collected using a standardized data collection form to ensure consistency and accuracy, which included demographic information such as age, gender, and residential location obtained through parental interviews and hospital records. Anthropometric measurements, including height and weight, were recorded using calibrated equipment, and BMI was calculated using the formula: BMI = weight (kg) /height^2^ (m^2^). Serum samples were collected to measure TC, TG, Ca, ALP, FBG and HbA1c in accordance with standard laboratory protocols. Data pre-processing involved detecting outliers using the interquartile range (IQR) method and handling missing values via multiple imputation techniques to ensure data integrity. To facilitate comparability between datasets from the two hospitals, *Z*-score normalization was performed on the biochemical parameters. The American Academy of Pediatrics’ criteria for vitamin D deficiency were applied: <15 ng/mL as deficiency, 15–20 ng/mL as insufficiency, and ≥20 ng/mL as sufficiency. In this study, participants were categorized into two groups based on their 25(OH)D_3_ serum levels: the deficiency group (*n* = 56), including both deficient (<15 ng/mL) and insufficient (15–20 ng/mL) levels; the non-deficiency group (*n* = 103), with sufficient levels (≥20 ng/mL).

### 2.2 Inclusion and exclusion criteria

The inclusion criteria were as follows: (1) All participants were children undergoing routine health examinations. (2) Age range of 6 to 12 years. (3) Availability of complete clinical data for each participant.

The exclusion criteria were as follows: (1) Physical growth abnormalities or deformities. (2) A history of gastrointestinal, respiratory, or other system-related illnesses and recent medication use within the past 3 months. (3) Immunological disorders or uncontrolled infections. (4) Impaired heart, liver, or kidney functions.

Ethics approval and consent to participate: From 2019 until late 2022, clinical data collected from 159 school-aged children during health examinations and outpatient visits at the Hainan Hospital of PLA General Hospital and Lingshui Li Autonomous County People’s Hospital. The Ethics Committee of Hainan Hospital of PLA General Hospital and the Ethics Committee of Lingshui Li Autonomous County People’s Hospital was responsible for supervising and approving the course of the entire study.

### 2.3 Collection of clinical blood samples

A total of 5 mL of venous blood was obtained from each child through standard venipuncture techniques, ensuring aseptic conditions. Blood samples were processed within 2 h post-collection to maintain the integrity of the biochemical analyses.

### 2.4 Measurement of TC and TG

TC and TG levels were measured using a ADVIA 2400 automated biochemical analyzer (Siemens AG, Germany). Serum was separated from whole blood via centrifugation at 3000 rpm for 10 min at room temperature. Serum samples are mixed with the corresponding reagents as per the manufacturer’s instructions, and the resulting TC and TG levels are assessed colorimetrically at 500 nm. All reagent kits are sourced from Roche (Shanghai, China).

### 2.5 Serum levels of FBG

FBG levels were assessed using the 7060 automated biochemical analyzer (Hitachi, Japan). Following serum preparation as described, the glucose oxidase method was utilized, wherein glucose is oxidized to gluconic acid and hydrogen peroxide. The resultant hydrogen peroxide is colorimetrically measured at 505 nm. The reagent kits are from Roche (Shanghai, China).

### 2.6 Serum levels of FBG

The levels of HbA1c were analyzed using the RT6000 microplate reader (Rayto, China). Whole blood was mixed with a buffer, incubated at room temperature for 15 min, and then centrifuged at 3,000 rpm. The supernatant was subsequently injected into the HPLC system, with measurements taken at an absorbance of 415 nm. Reagent kits were supplied by Roche (Shanghai, China).

### 2.7 Serum levels of calcium and ALP

Calcium and ALP levels were measured using the Hitachi 7060 automated biochemical analyzer. After centrifugation of serum at 4°C, serum samples were mixed with the reagents for calcium and ALP assays in accordance with Roche (Shanghai, China) instructions. The calcium levels were measured at 650 nm, while the ALP levels were assessed at 405 nm.

### 2.8 Measurement of 25(OH)D_3_ levels

The measurement of 25(OH)D_3_ levels was performed using an enzyme-linked immunosorbent assay (ELISA) by Roche (Shanghai, China). Serum samples were diluted in a buffer, followed by the addition of specific antibodies against 25(OH)D_3_. After incubation and washing, a substrate solution was added to produce a color change proportional to the 25(OH)D_3_ concentration in the sample. Absorbance was measured at 450 nm using the 680 microplate reader (Bio-Rad, United States).

### 2.9 Bone age assessment and calculation of body mass index

All children underwent a radiographic examination of the left wrist in the hospital after admission using a Shimadzu X-ray machine. During the radiographic examination, the palm and fingers were placed facing downwards on the detector. The children were instructed by the physician to extend their forearms and hands until the forearm axis aligned with the middle finger axis, with their fingers naturally spread apart. The central axis of the X-ray beam was aligned with the third metacarpophalangeal joint gap, and a perpendicular projection was taken, with the focal-film distance set at 90 cm. Furthermore, the BMI was calculated following the collection of admission statistics for the children, using the formula BMI = body weight (kg) /height^2^ (m^2^).

### 2.10 Statistical analysis

Data analysis was performed using SPSS 22.0. Continuous variables were assessed for normality using the Kolmogorov–Smirnov test. Independent sample *t*-tests were employed for normally distributed variables, while non-normally distributed data were analyzed using the Mann–Whitney *U* test. Categorical data were compared using the *χ*^2^ test. Spearman correlation analysis was performed to explore associations between 25(OH)D_3_ levels and BMI, TC, TG, Ca, ALP, and bone age. Multivariate logistic regression analysis was conducted to assess the independent predictors of 25(OH)D_3_ deficiency. ROC curve analysis was used to evaluate the predictive value of BMI, TC, TG, Ca, ALP, and bone age in diagnosing vitamin D deficiency. Statistical significance was set at *p* < 0.05.

## 3 Results

### 3.1 Basic information of the two groups and comparison of laboratory indices and bone age between the two groups

**Figure 1:**
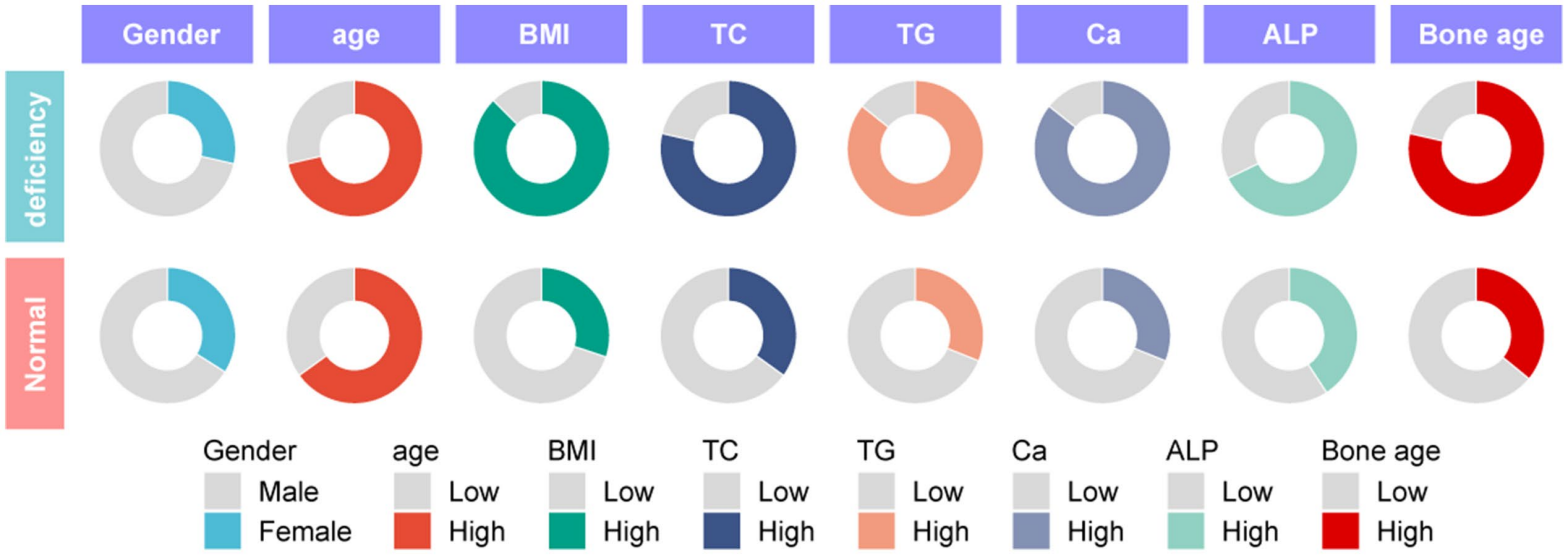
Patient baseline characteristics diagram.

**Figure 2:**
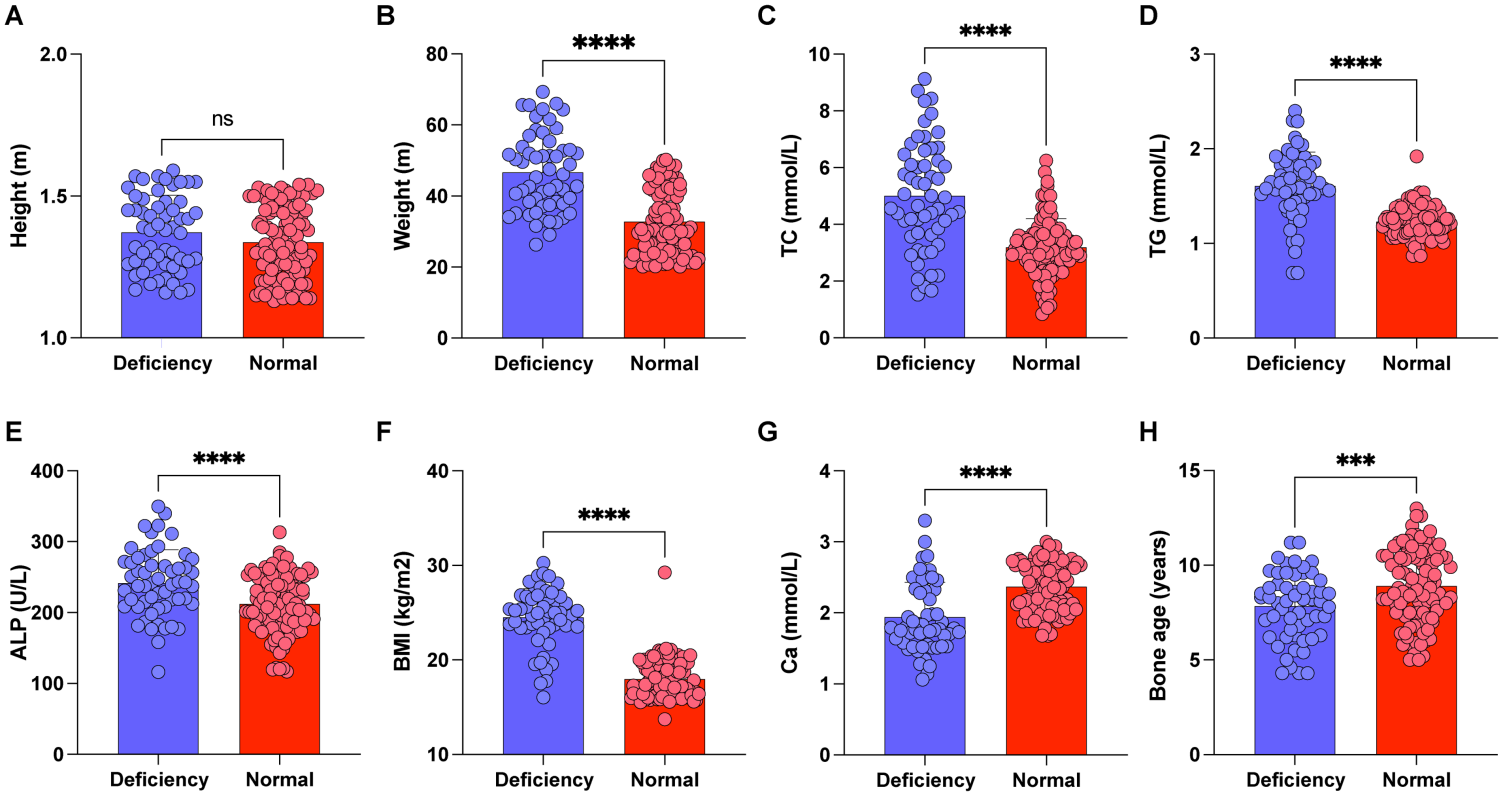
Comparison of height, weight, BMI, TC, TG, Ca, ALP and bone age between 25-(OH)D_3_ deficiency group and non-25-(OH)D_3_ deficiency group. (A) Height. (B) Weight. (C) TC. (D) TG. (E) Ca. (F) BMI. (G) ALP. (H) Bone age. ns *p* > 0.05, ^***^p < 0.001, ^***^p < 0.0001.

To explore the correlation between vitamin D deficiency and the prevalence of obesity in children, we conducted a retrospective analysis of the clinical data from 159 school-age children who underwent physical examinations (Figure 1). Among the participants, 109 were male and 50 were female, with ages ranging from 6 to 12 years and an average age of 9.27 ± 2.10 years. No significant differences were observed in gender, age, height (Figure 2A), FBG, and HbA1c between the two cohorts (*p* > 0.05). However, the group with 25(OH)D_3_ deficiency showed significantly higher levels of weight, BMI, TC, TG, and ALP compared to the non-deficient group (Figures 2B–F). In contrast, Ca levels and bone age were significantly lower in the 25(OH)D_3_ deficiency group (Figures 2G,H). The general characteristics of these patients are detailed in Table 1.

**Table 1:** Baseline characterization of participation.

Item	25-(OH)D_3_ deficiency group (*n* = 56)	Non-25-(OH)D_3_ deficiency group (*n* = 103)	*x^2^*/*t*	*P*
Gender (*n*)			0.01	0.88
Male [*n* (%)]	38	71		
Female [*n* (%)]	18	32		
Age (years)	9.24 ± 2.06	9.30 ± 2.11	0.17	0.86
Height (m)	1.36 ± 0.11	1.31 ± 0.11	5.23	0.1
Weight (kg)	48.60 ± 9.37	32.09 ± 7.23	8.83	<0.001
BMI (kg/m^2^)	24.10 ± 3.12	17.96 ± 1.28	9.62	<0.001
TC (mmol/L)	4.92 ± 1.88	3.18 ± 1.07	7.44	<0.001
TG (mmol/L)	1.62 ± 0.39	1.23 ± 0.14	9.14	<0.001
FBG (mmol/L)	4.23 ± 1.01	4.24 ± 1.06	0.05	0.95
HbA1c (%)	4.90 ± 0.67	4.88 ± 0.63	0.18	0.85
Ca (mmol/L)	1.94 ± 0.15	2.64 ± 0.21	22.05	<0.001
ALP (U/L)	241.50 ± 61.27	212.72 ± 46.85	3.31	<0.001
Bone age (years)	6.18 ± 1.36	7.58 ± 1.51	5.77	<0.001

### 3.2 Comparison of left wrist normal-position X-rays in the two groups of children

**Figure 3:**
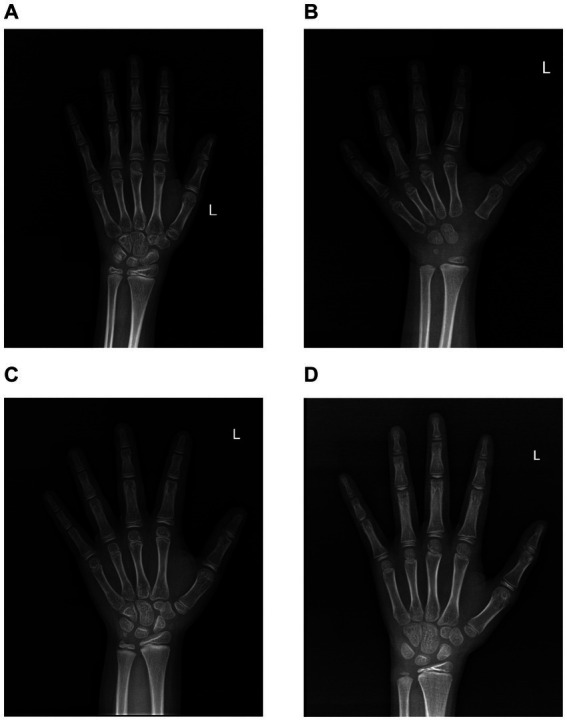
Images of normal-position X-rays of the left wrist in the two cohorts. (A) The normal-position X-ray image of the left wrist in a 9-year-old female from the 25(OH)D_3_ normal group. (B) The normal-position X-ray image of the left wrist in a 9-year-old male from the 25(OH)D_3_ normal group. (C) The normal-position X-ray image of the left wrist in a 9-year-old male from the 25(OH)D_3_ insufficiency group. (D) The normal-position X-ray image of the left wrist in a 9-year-old male from the 25(OH)D_3_ deficiency group.

In the present investigation, we collected anteroposterior radiographs of the left hand from a cohort of school-age children for bone age evaluation. Furthermore, we have featured a subset of exemplary radiographic findings. Figure 3A presents a standard anteroposterior X-ray image of the left wrist from a 9-year-old female participant in the non-25(OH)D_3_ group. The radiographic R-series indicates a bone age advancement of 9 months relative to chronological age, while the C-series demonstrates a normal bone age alignment. Figure 3B likewise represents an anteroposterior radiograph of the left wrist from a 9-year-old male, the R-series suggests a bone age 5 months older than the actual age, and the C-series indicates a 1-year and 2-month difference. Figure 3C shows a radiograph from a 9-year-old male exhibiting 25(OH)D_3_ insufficiency. The R series indicates a bone age 2 years younger than his actual age, while the C series suggests a bone age 1 year and 10 months older. Similarly, we found that school-aged children within the 25(OH)D_3_ deficiency cohort exhibit lower R-series and C-series ages than their peers (Figure 3D). In summary, our research findings indicate that a deficiency in 25(OH)D_3_ during the school-age period is a significant risk factor for delayed growth and development.

### 3.3 The value of BMI, TC, TG, Ca, ALP, and bone age in predicting 25(OH)D_3_ deficiency in school-aged children

**Figure 4:**
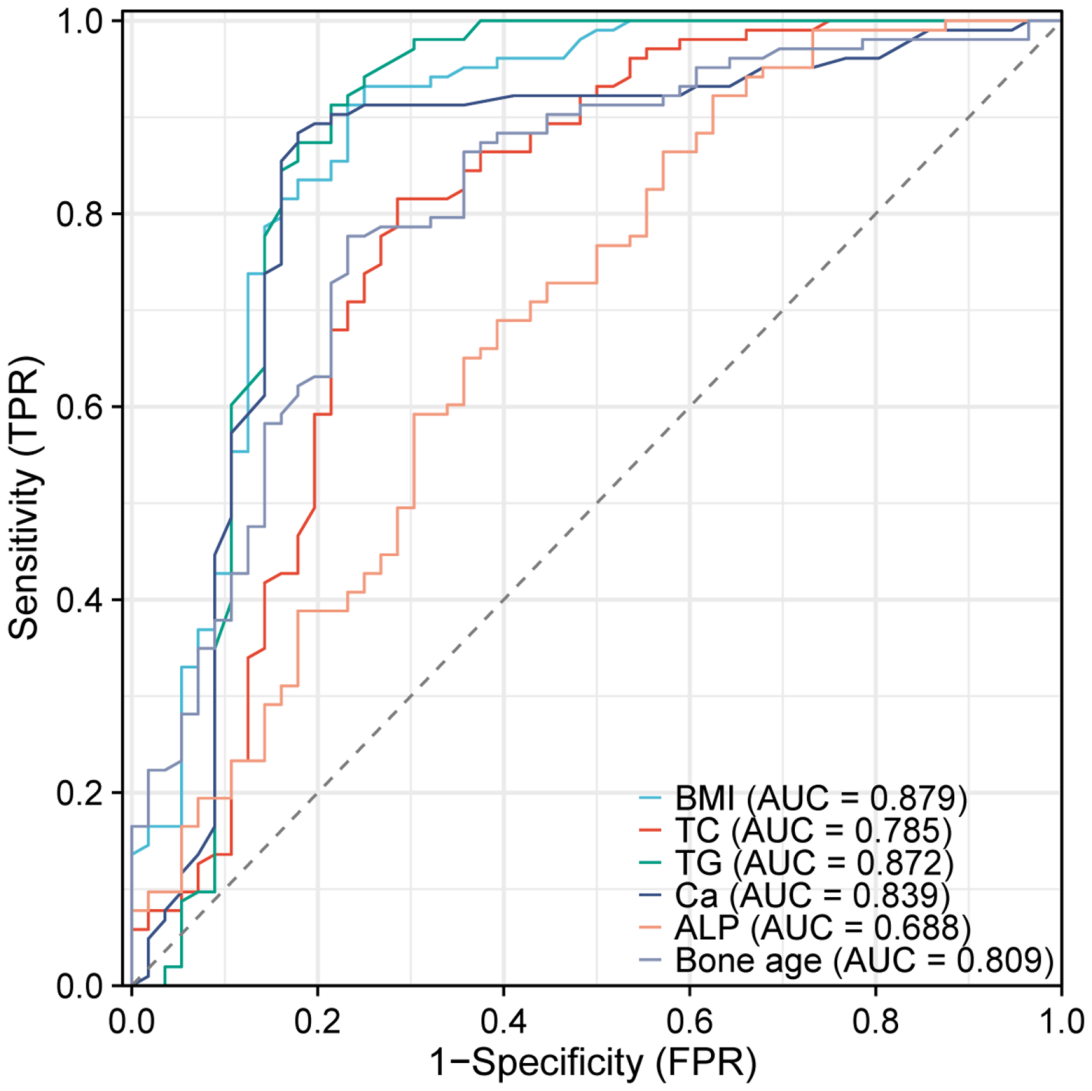
ROC curves of BMI, TC, TG, Ca, ALP, and bone age predicting 25(OH)D_3_ deficiency in school-aged children.

We further constructed the receiver operating characteristic (ROC) curve to assess whether BMI, TC, TG, Ca, ALP, and bone age could be utilized for forecasting 25(OH)D_3_ deficiency in school-aged children. The AUC for each parameter was BMI (AUC: 0.879), TC (AUC: 0.785), TG (AUC: 0.872), Ca (AUC: 0.839), ALP (AUC: 0.688), and bone age (AUC: 0.809), respectively, all of which presented a significance level of *p* < 0.05 (Table 2). The results confirmed that a vast majority of clinical Indicators have a significant diagnostic value (Figure 4).

**Table 2:** The value of BMI, TC, TG, Ca, ALP, and bone age in predicting 25-(OH)D_3_ deficiency in school-aged children.

Index	AUC	SE	*p*	95% CI	Optimum cutoff value	Sensitivity	Specificity
BMI	0.879	0.815–0.943	0.682	24.50 kg/m^2^	0.932	0.750	0.879
TC	0.785	0.700–0.868	0.530	4.08 mmol/L	0.816	0.714	0.785
TG	0.872	0.797–0.948	0.698	1.39 mmol/L	0.913	0.785	0.872
Ca	0.839	0.762–0.915	0.704	2.18 mmol/L	0.884	0.821	0.839
ALP	0.688	0.598–0.778	0.297	306.26 U/L	0.922	0.375	0.688
Bone age	0.809	0.736–0.881	0.545	7.25 years	0.777	0.768	0.809

### 3.4 Analysis of the relationship between 25(OH)D_3_ levels and BMI, TC, TG, Ca, ALP, and bone age

**Figure 5:**
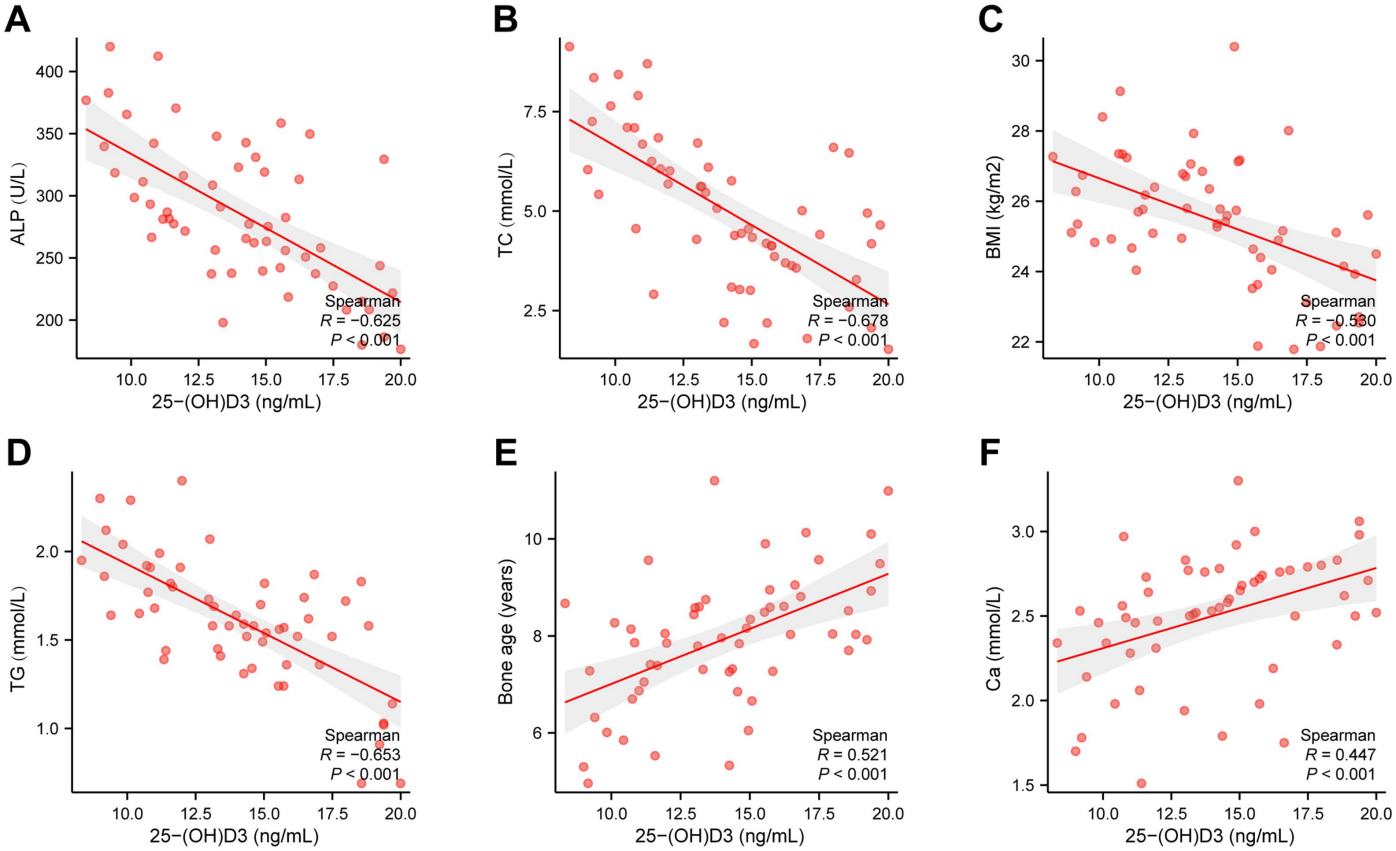
Spearman’s correlation analysis of the relationship between 25(OH)D_3_ levels and BMI, TC, TG, Ca, ALP, and bone age. (A) ALP (*R*^2^ = −0.6019, *p* < 0.0001). (B) TC (*R*^2^ = −0.5998, *p* < 0.0001). (C) BMI (*R*^2^ = −0.6234, *p* < 0.0001). (D) TG (*R*^2^ = −0.6125, *p* < 0.0001). (E) Bone age (*R*^2^ = 0.6729, *p* < 0.0001). (F) Ca (*R*^2^ = 0.5920, *p* < 0.0001).

To further analyze whether there is a direct correlation between 25(OH)D_3_ levels and BMI, BMI, TC, TG, Ca, ALP, and bone age. We selected 56 children from the 25(OH)D_3_ deficiency cohort and used Spearman’s correlation analysis to assess whether there is an association between their serum 25(OH)D_3_ levels and BMI, TC, TG, Ca, ALP, and bone age. The outcomes illustrated that there was a negative correlation between 25(OH)D_3_ levels in the children’s serum and BMI (*R*^2^ = −0.6234, *p* < 0.0001), TC (*R*^2^ = −0.5998, *p* < 0.0001), TG (*R*^2^ = −0.6125, *p* < 0.0001), and ALP (*R*^2^ = −0.6019, *p* < 0.0001) (Figures 5A–D), whereas a positive correlation was observed with Ca (*R*^2^ = 0.5920, *p* < 0.0001) and bone age (*R*^2^ = 0.6729, *p* < 0.0001) (Figures 5E–F). These correlations were statistically significant with *p* < 0.05, as presented in Table 3.

**Table 3:** Analysis of the relationship between 25-(OH)D_3_ levels and outcomes.

Index	*R* ^2^	*p*
BMI	−0.530	<0.001
TC	−0.678	<0.001
TG	−0.653	<0.001
Ca	0.447	<0.001
ALP	−0.625	0.009
Bone age	0.521	<0.001

**Figure 6:**
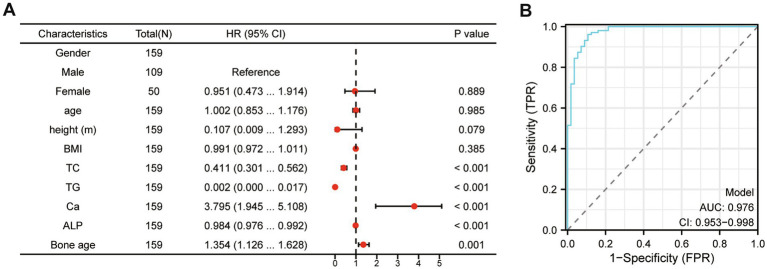
The logistic regression and multivariate ROC curve comprehensive analysis of the relationship between 25(OH)D_3_ levels and BMI, TC, TG, Ca, ALP, and bone age. (A) The logistic regression analysis. (B) Multivariate ROC curve comprehensive analysis (BMI, TC, TG, Ca, ALP, and bone age).

To further explore the relationship between 25(OH)D_3_ levels and BMI, TC, TG, Ca, ALP, and bone age, we performed a logistic regression analysis. The dependent variable was the presence of 25(OH)D_3_ deficiency, while BMI, TC, TG, Ca, ALP, and bone age were included as independent variables in the model. The logistic regression analysis revealed that Ca, ALP levels and bone age were significant independent predictors of 25(OH)D_3_ deficiency, with odds ratios (ORs) greater than 1 (*p* < 0.05), indicating an increased risk of deficiency (Figure 6A). To further substantiate our findings, we developed a ROC curve using multiple indicators (BMI, TC, TG, Ca, ALP, and bone age), which demonstrated the strong predictive value of these factors for identifying 25(OH)D_3_ deficiency (Figure 6B). These findings further support the significant correlations observed in the Spearman analysis, demonstrating the strength of association between these metabolic and bone-related markers and 25(OH)D_3_ deficiency.

### 3.5 Subgroup analysis based on BMI in 25(OH)D_3_ deficient children

**Figure 7:**
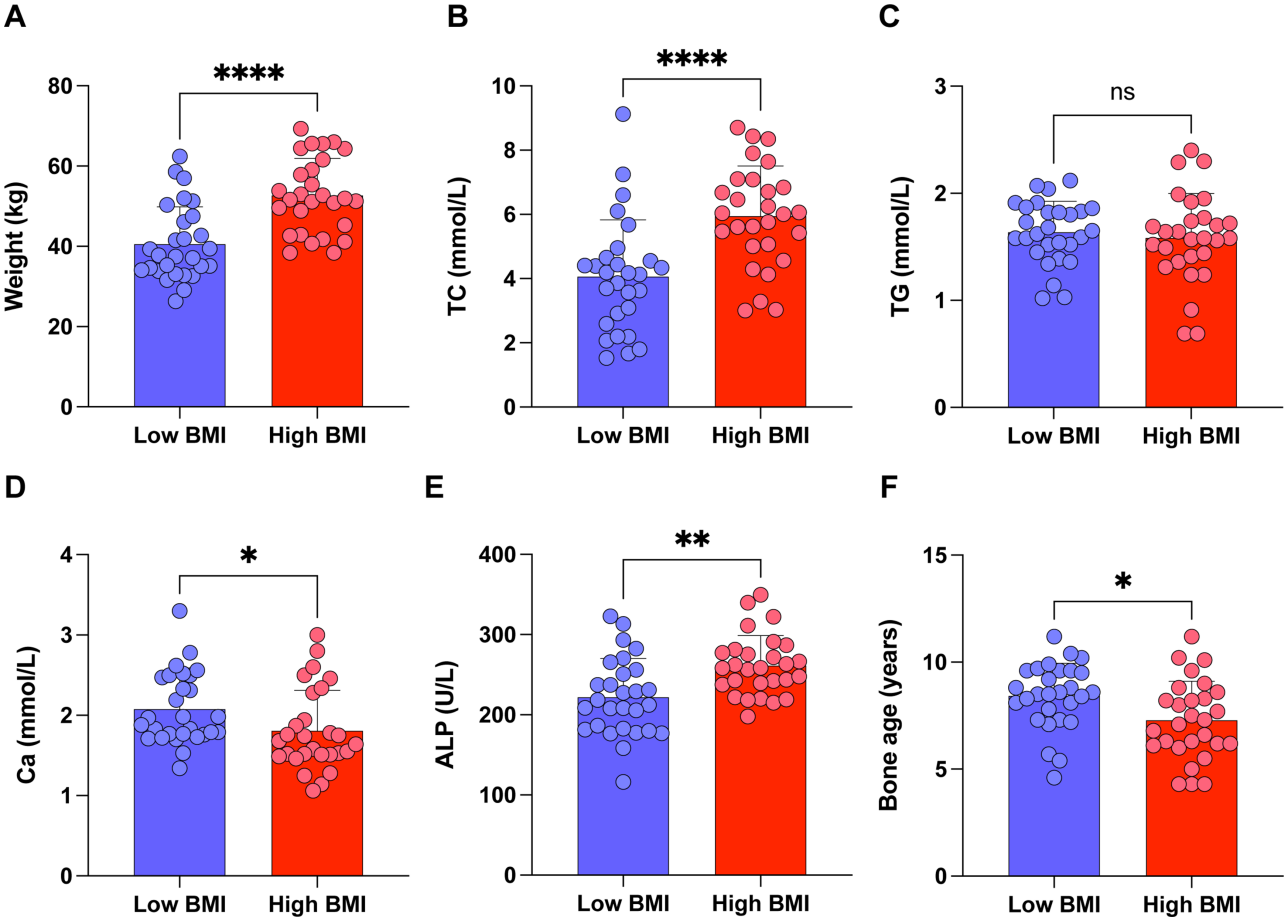
Subgroup analysis of children with 25(OH)D_3_ deficiency stratified by BMI levels. (A) Weight. (B) TC. (C) TG. (D) Ca. (E) ALP. (F) Bone age. ns *p* > 0.05, ^*^p < 0.05, ^**^p < 0.01, and ^****^p < 0.0001.

Based on our findings, vitamin D deficiency is closely linked to obesity in school-age children. We further conducted a subgroup analysis of children with 25(OH)D_3_ deficiency stratified by their BMI levels. Our analysis revealed that 25(OH)D_3_ deficient children with higher BMI had significantly elevated levels of TC, ALP, and advanced bone age, while Ca levels were relatively lower (Figures 7A–F). These findings highlight a stronger association between obesity and specific metabolic and bone-related markers in children with 25(OH)D_3_ deficiency, providing further insight into the complex interplay between 25(OH)D_3_ status and childhood obesity.

## 4 Discussion

Over the last several years, with the improvement in people’s quality of life, childhood obesity has become increasingly prominent. As opposed to healthy children, this group shows a dramatic elevation in BMI, and the risk of cardiovascular and metabolic diseases is also vigorously heightened (12–14). 25(OH)D_3_, as a common indicator for evaluating vitamin D, plays a crucial role in regulating Ca levels and influencing cell proliferation and differentiation, which are of great significance in the body’s growth and development. Therefore, this research seeks to probe the relationship between 25(OH)D_3_ and BMI, TC, and TG in school-aged children, observing the impact of alterations in 25(OH)D_3_ levels on childhood obesity. In addition, Ca, ALP, and bone age are important indicators of bone metabolism. Actively investigating the influence of 25(OH)D_3_ changes on Ca, ALP, and bone age may provide a theoretical basis for understanding the abnormalities in Ca, ALP, and bone age caused by vitamin D deficiency in school-aged children.

The findings of this study suggested that the 25(OH)D_3_ deficiency cohort had substantially heightened BMI, TC, and TG levels compared to the non-deficiency cohort, which meant that 25(OH)D_3_ deficiency could culminate in childhood obesity. In a preceding study, researchers have ascertained that 25(OH)D_3_ in obese children is vigorously lowered vis-à-vis normal-weight children. In this scenario, the BMI of obese children abnormally increases. They have also pointed out that with the increasing age of obese children, the 25(OH)D_3_ deficiency worsens, which can mutually support the findings of this study (15). TC and TG are both commonly utilized clinical indicators for assessing the lipid content within the blood. The former reflects the total cholesterol contained in lipoproteins in the blood, while the latter reflects the total triglycerides contained in lipoproteins. An uplift in their levels denotes fat accumulation within the body (16, 17). 25(OH)D_3_ assumes a function in augmenting calcium ion levels within fat cells and fatty acid synthetase activity, making itself an indispensable inhibitor during the process of fat cell differentiation. When there is a deficiency of 25(OH)D_3_ in school-aged children, the inhibitory effect of 25(OH)D_3_ is affected, resulting in fat accumulation within the body and abnormally elevated BMI, TC, and TG levels (18, 19). It is worth noting that in the current research, FBG and HbA1c serum levels in both cohorts did not exhibit remarkable disparities, revealing that 25(OH)D_3_ deficiency in school-aged children might not have a huge impact on glucometabolic. Nonetheless, in prior research, Safarpour et al. (20) have unraveled that vitamin D supplementation can achieve FBG and HbA1c modulation. Corica et al. (21) have also confirmed that vitamin D deficiency in obese children can bring about impaired glucose metabolism and elevated blood glucose levels. The divergences between the above-mentioned reports and the findings of this study may be attributed to factors such as differences in the age and geographical location of the study subjects.

Our research also unveiled that the 25(OH)D_3_ deficiency cohort had significantly lower levels of Ca, ALP, and bone age vis-a-vis the non-deficiency cohort, suggesting that 25(OH)D_3_ insufficiency impinged on the bone growth and development of school-aged children. Vitamin D is an essential humoral factor that modulates bone metabolism and sustains normal development in the body. It can be obtained through sunlight exposure, UV radiation, and dietary intake. On one hand, vitamin D stimulates the production of intestinal calcium-binding proteins, augments blood Ca levels, and boosts bone mineralization. On the other hand, vitamin D induces the maturation and differentiation of osteoblasts, facilitating the formation and maturation of bone matrix (22–24). In cases of vitamin D deficiency, only 10–15% of dietary calcium can be absorbed due to the lack of calcium within the serum, giving rise to an imbalance in the calcium-phosphorus ratio, which disrupts normal epiphyseal cartilage growth and mineralization, leading to growth retardation and bone deformities. Thus, in situations of vitamin D deficiency, the supplementation of calcium alone has minimal effect on growth and development and may even cause bone deformities such as epiphyseal protrusion and rib beading (25). ALP is an enzyme present in multiple tissues throughout the body, originating from the liver, bones, intestines, or kidneys. However, bone ALP and liver ALP account for approximately 95% of the total ALP activity in human serum. Research has unveiled that the most common cause of heightened ALP levels in the blood is related to liver or bone diseases (26). ALP exerts a critical function in modulating calcium absorption and utilization through its involvement in mineral transformation in the bones and the activation of vitamin D. In bones, ALP steps up the release of phosphate ions and forms minerals in combination with calcium ions, maintaining bone structure and strength. Furthermore, the activity level of ALP can reflect alterations in calcium and phosphorus metabolism. When there is an imbalance in calcium and phosphorus metabolism, such as inadequate calcium absorption or excessive excretion, the activity of ALP often undergoes changes. Therefore, by monitoring ALP activity, the status of calcium and phosphorus metabolism can be assessed, providing further insights into bone health. This study further demonstrated that the 25(OH)D_3_ deficiency cohort displayed a dramatic elevation in ALP, and the reason for this is associated with the high bone turnover state resulting from vitamin D insufficiency. Due to the impact of vitamin D deficiency, there is an imbalance in the calcium-phosphorus proportion, culminating in impaired bone growth and development. In this state, osteoblasts become more active, which can cause a rise in the ALP level. Bellastella et al. (27) have also pinpointed in their research that serum ALP profile is modulated by vitamin D, denoting a close correlation between the two.

In our research, the value of BMI, TC, TG, Ca, ALP, and bone age in forecasting 25(OH)D_3_ insufficiency in school-aged children was observed through ROC analysis. It was discovered that BMI ≥24.50 kg/m^2^, TC ≥4.08 mmol/L, TG ≥1.39 mmol/L, Ca ≤2.18 mmol/L, ALP ≤306.26 U/L, and bone age ≤7.25 years are indicators with high sensitivity for predicting 25(OH)D_3_ deficiency. Actively monitoring these indicators may provide assistance in early identification of 25(OH)D_3_ deficiency in school-aged children. Additionally, this research investigated the correlation between 25(OH)D_3_ levels and BMI, TC, TG, Ca, ALP, and bone age in school-aged children through the assistance of Spearman coefficients. It was confirmed that 25(OH)D_3_ levels in school-aged children were negatively associated with BMI, TC, TG, and ALP but positively correlated with Ca and bone age. This unveiled a close relationship between 25(OH)D_3_ deficiency and childhood obesity as well as bone growth and development. In clinical practice, particular attention should be devoted to monitoring the fluctuations in 25(OH)D_3_ levels among school-aged children, ensuring their optimal growth and development during this critical period.

Nonetheless, our study is subject to certain limitations. Firstly, while the two hospitals participating in this research are situated within the same geographical region—thereby reducing potential bias from geographic disparities—the relatively small sample size may still introduce a degree of bias into the final statistical results. Secondly, the obesity-related indicators analyzed were somewhat limited; due to incomplete data, specific metrics such as waist circumference could not be further examined. In future investigations, we intend to conduct multicenter collaborative analyses that encompass a wider array of relevant indicators and delve deeper into the mechanisms by which 25(OH)D_3_ affects obesity and growth retardation in school-aged children. These initiatives will significantly contribute to enhancing health management strategies for this demographic.

## 5 Conclusion

In conclusion, it can be inferred that insufficiency of 25(OH)D_3_ may contribute to school-age children’s obesity. Furthermore, this deficiency exerts an impact on bone growth and development in these children, leading to reduced levels of calcium, delayed bone maturation, and ALP levels. When conducting clinical work, prioritizing the screening for vitamin D deficiency in school-age children is particularly crucial.

## Data Availability

The original contributions presented in the study are included in the article/supplementary material, further inquiries can be directed to the corresponding author.
